# Brain aging in major depressive disorder

**DOI:** 10.1192/j.eurpsy.2021.196

**Published:** 2021-08-13

**Authors:** L. Han, H. Schnack, R. Brouwer, D. Veltman, N. Van Der Wee, M.-J. Van Tol, M. Aghajani, B. Penninx

**Affiliations:** 1 Department Of Psychiatry, Amsterdam University Medical Centers, Vrije Universiteit and GGZ inGeest, Amsterdam Neuroscience, Amsterdam, Netherlands; 2 Department Of Psychiatry, UMCU Brain Center, University Medical Center Utrecht, Utrecht, Netherlands; 3 Department Of Psychiatry, Leiden University Medical Center, Leiden, Netherlands; 4 Cognitive Neuroscience Center, University Medical Center Groningen, University of Groningen, Groningen, Groningen, Netherlands

**Keywords:** Depression, brain age, antidepressant use, Anxiety

## Abstract

Depression and anxiety are common and often comorbid mental health disorders that represent risk factors for aging-related conditions. Brain aging has shown to be more advanced in patients with Major Depressive Disorder (MDD). Here, we extend prior work by investigating multivariate brain aging in patients with MDD and/or anxiety disorders and examine which factors contribute to older appearing brains. Adults aged 18-57 years from the Netherlands Study of Depression and Anxiety underwent structural MRI. A pre-trained brain age prediction model based on >2,000 samples from the ENIGMA consortium was applied to obtain brain-predicted age differences (brain-PAD, predicted brain age minus chronological age) in 65 controls and 220 patients with current MDD and/or anxiety. Brain-PAD estimates were associated with clinical, somatic, lifestyle, and biological factors. After correcting for antidepressant use, brain-PAD was significantly higher in MDD (+2.78 years, Cohen’s d=0.25, 95% CI -0.10-0.60) and anxiety patients (+2.91 years, Cohen’s d=0.27, 95% CI -0.08-0.61), compared to controls. There were no significant associations with lifestyle or biological stress systems. A multivariable model indicated unique contributions of higher severity of somatic depression symptoms (b=4.21 years per unit increase on average sum score) and antidepressant use (-2.53 years) to brain-PAD. Advanced brain aging in patients with MDD and anxiety was most strongly associated with somatic depressive symptomatology. We also present clinically relevant evidence for a potential neuroprotective antidepressant effect on the brain-PAD metric that requires follow-up in future research.

**Disclosure:**

No significant relationships.

